# Direct Space Structure Solution Applications

**DOI:** 10.6028/jres.109.004

**Published:** 2004-02-01

**Authors:** Maryjane Tremayne

**Affiliations:** School of Chemistry, University of Birmingham, Edgbaston, Birmingham, B15 2TT, UK

**Keywords:** chlorothalonil, cocrystals, crystal engineering, direct-space methods, hydrogen bonding, Monte Carlo, nicotinic acid, polymorphism, powder diffraction, preferred orientation, structure solution, sulfonamide

## Abstract

The crystal structures of 2,4,6-triisopropylbenzenesulfonamide, 1,2,3-trihydroxybenzene-hexamethylenetetramine (1/1), 5-bromonicotinic acid and chlorothalonil form II have been solved from x-ray powder diffraction data, by application of a direct space structure solution approach using the Monte Carlo method and confirmed by Rietveld refinement. In the sulfonamide, the molecules are linked by N–H⋯O hydrogen bonds into two-dimensional sheets built from alternating eight and twenty-membered rings. In the cocrystal, the molecules are linked by O–H⋯N hydrogen bonds to form puckered molecular ribbons that are in turn linked into a continuous 3D framework by C–H⋯π (arene) interactions. 5-bromonicotinic acid also displays atypical hydrogen-bonding behaviour by formation of dimers through a self-complementary acid-acid hydrogen-bond motif that are connected into antiparallel ribbons by C–H⋯O and C–H⋯N hydrogen bonds. Structure determination of the cocrystal and the bromonicotinic acid was successful despite the presence of preferred orientation in the data, whereas the distortion of the chlorothalonil data was so severe that structure solution was only possible when the effects of preferred orientation were minimized. Both the disordered structure, and an ordered structural approximation of chlorothalonil form II have been determined and rationalized.

## 1. Introduction

The *ab initio* crystal structure determination of molecular materials from x-ray powder diffraction data is a rapidly expanding field, and has grown substantially in the last few years mainly due to the development and application of new methods of structure solution, in particular “direct-space” based techniques [1,2]. These methods approach structure solution by generation of trial crystal structures based on the known molecular connectivity of the material. The fitness of each structure is then assessed by comparison of the corresponding calculated diffraction pattern and the experimental diffraction data. Global optimization techniques such as Monte Carlo [3–8], simulated annealing [9–13] or genetic algorithms [14–18] are used to locate the global minimum corresponding to the best structure solution.

In this paper, we present a number of organic structure solution problems that have been resolved from conventional laboratory and synchrotron powder diffraction data using a direct-space structure solution technique based on the Metropolis Monte Carlo algorithm [19] and implemented in the program POSSUM [20]. The compounds studied are selected from two main areas of our research; the study of hydrogen-bond networks and polymorphism, and consist of single and multi-component systems containing both rigid and conformationally flexible molecules.

A number of these structures have been determined from powder data significantly affected by preferred orientation; a sample characteristic that arises when crystallites have a tendency to align along a certain direction resulting in a non-random distribution of crystallite orientations in the sample, affecting the relative intensities of given peaks. This distortion of the data can have a disastrous effect on traditional structure solution, whereas direct-space methods appear to be more robust, presumably because a substantial amount of structural knowledge is included in the calculation through the use of a structural model. However, in severe cases (i.e., when the morphology is strongly anisotropic) we illustrate that the direct-space structure solution of even simple structural problems can fail.

## 2. Crystal Engineering and the Study of Intermolecular Interactions

In organic molecular crystals, hydrogen bonds often constitute the strongest intermolecular synthon [21], and hence often dictate the preferred packing arrangement of the molecules. The general principles underlying the formation of hydrogen bonds are reasonably well understood, but there are at present few, if any, reliable methods for the prediction of hydrogen-bonding patterns. A detailed description of the hydrogen-bonding patterns in a given system must be derived from analysis of specific experimental data, and as such, a sound knowledge and understanding of the role that intermolecular forces play in supramolecular assembly is generally obtained from systematic crystallographic studies. Materials of interest in this field are ideal targets for direct-space structure solution techniques, particularly when the structures consist of well-defined molecular building blocks, with the intermolecular aggregation of these building blocks within the crystal structure being of primary interest. In this section we highlight the contribution of direct-space structure solution methods to a number of systematic structural studies, including a long-standing investigation of a family of sulfonylamino compounds entirely from powder diffraction data, the study of atypical crystal packing in a group of nicotinic acid derivatives and report the first example of powder diffraction being used in the structure solution of an organic cocrystal.

### 2.1 Sulfonylamino and Related Compounds

**Scheme 1:**
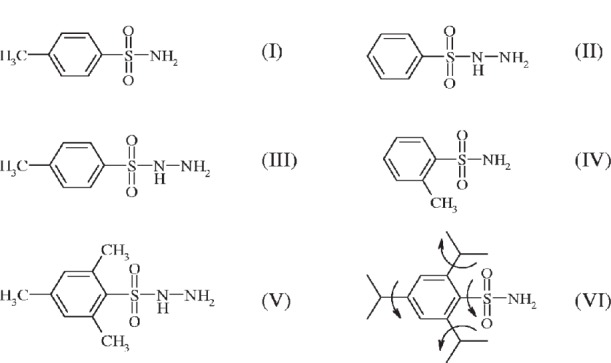


In previous work [22], we have reported the *ab initio* structure determination of three sulfonylamino compounds, using powder x-ray diffraction data collected using a conventional laboratory powder diffractometer (Scheme 1). The structures of 4-toluenesulfonamide CH_3_C_6_H_4_SO_2_NH_2_ (I) and benzenesulfonylhydrazine C_6_H_5_SO_2_NHNH_2_ (II) were readily solved using traditional direct methods programs, while the structure of 4-toluenesulfonylhydrazine CH_3_C_6_H_4_SO_2_NHNH_2_ (III) was solved using the maximum entropy and likelihood method MICE [23]. Similar data sets were recorded for 2-toluenesulfonamide CH_3_C_6_H_4_SO_2_NH_2_ (IV), 2, 4, 6 – trimethyl benzenesulfonyl hydrazine (Me)_3_C_6_H_2_SO_2_NHNH_2_ (V), and 2,4,6-tri-isopropylbenzenesulfonamide (Me_2_CH)_3_C_6_H_2_SO_2_NH_2_ (VI). Although these data enabled indexing of (IV) and (VI), attempts at structure solution by traditional methods were unsuccessful. The diffraction pattern of (V) could not be indexed from the data available. A new low-temperature data set for (VI) was collected using synchrotron x-ray radiation, and details of the crystal structure determination by the Monte Carlo method are given below.

#### 2.1.1 Structure Determination of 2,4,6-Tri-Isopropylbenzenesulfonamide

The new data set was collected at station 2.3 of the SRS, Daresbury Laboratory at a temperature of 120(1) K, and indexed giving a unit cell similar to that obtained from the corresponding ambient-temperature laboratory data [5]. Structure solution was carried out using the Monte Carlo method with a structural model comprising the complete molecule excluding the methyl hydrogen atoms, and constructed using standard bond lengths and angles. Although the benzene ring was maintained as a rigid body, the three isopropyl groups and the sulfonamide group were allowed to rotate freely and independently within the molecule as shown in Scheme 1. The initial position, orientation, and intramolecular geometry of the structural fragment were chosen arbitrarily and the random movement of the molecule in the Monte Carlo calculation carried out by translation and rotation of the structural fragment within the unit cell, simultaneously with the intramolecular rotations. After a sufficient amount of parameter space had been searched, the best structure solution was then taken as the starting model for Rietveld refinement (Table 1). The positions of all atoms were refined subject to soft restraints on the standard geometric parameters and the methyl H atoms were added to the molecule in positions consistent with standard geometry. Isotropic atomic displacement parameters were refined for the non-hydrogen atoms, but were constrained according to atom type or environment, i.e., S, O, or N; aromatic, propyl (CHMe_2_) or methyl C. The amino H atoms were placed in positions calculated from the coordinates of the hydrogen-bond donor and acceptors, but had no effect whatsoever on the refinement.

#### 2.1.2 Hydrogen Bonding and Molecular Conformation

The structure of (VI) is built from discrete molecules linked together by N–H⋯O hydrogen bonds. The conformation of the isopropyl groups is such that the isopropyl C–H bonds all lie approximately parallel to the plane of the aryl ring, with the methyl substituents indicative of repulsive interactions between the isopropyl groups and the sulfonamido group. This conformation of the three independent isopropyl groups appears to be the norm for 2,4,6-tri-isopropyl species (Me_2_CH)_3_C_6_H_2_X regardless of the identity of the *α*-atoms in the substituent X. In nearly all previously reported examples (see Refs. in [5]), the 2,4,6-tri-isopropylphenyl group was employed simply as a sterically bulky blocking group to protect some other part of the molecule, and none of these structure reports comment on its conformation. However, our analysis shows that the conformation of the isopropyl groups is essentially the same in all cases.

**Fig. 1:**
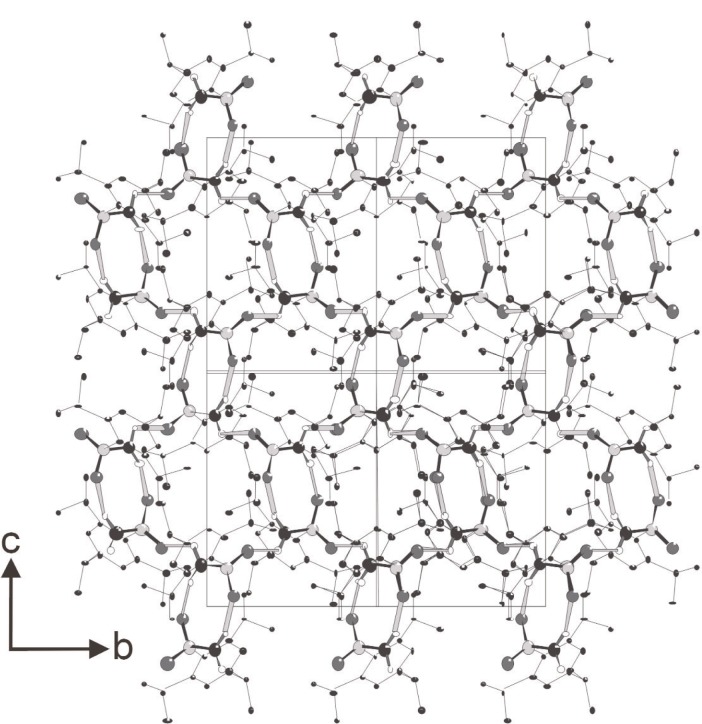
The crystal structure of (VI). Hydrogen-bonded motifs combine to form a sheet with 8-membered and 20-membered rings in a checkerboard pattern. Thin lines represent bonds to C, lines of intermediate thickness represent covalent S–N or S=O bonds, and thick lines represent N–H⋯O=S hydrogen bonds.

The NH_2_ group in (VI) acts as a double donor of hydrogen bonds, with a sulfone oxygen in each of two different molecules acting as the acceptors. These interactions result in formation of C(4) spirals, based on the N–H⋯O=S motif and generated by 2_1_ screw axes, and the generation of a cyclic R^2^_2_(8) motif around the centres of inversion (Fig. 1). The C(4) motif of N–H⋯O=S hydrogen bonds is extremely common in sulfonamides [5,22], and the R^2^_2_(8) motif has also been observed in sulfonamides [24,25], but these two motifs do not normally occur together in a single sulfonamide. The R^2^_2_(8) rings have the effect of linking together two adjacent but anti-parallel C(4) spirals. The propagation of these two hydrogen-bond motifs by means of the combined action of 2_1_ screw axes and centres of inversion leads to the generation of a continuous two-dimensional sheet parallel to (100) in which R^2^_2_(8) and R^6^_6_(20) rings alternate in a checkerboard pattern (Fig. 1). The tri-isopropylphenyl units lie on either side of the hydrogen-bonded sheet, so that the overall structure is that of a sandwich: a polar layer containing only S, O, N and H atoms lies between two non-polar hydrocarbon layers with only van der Waals contacts between adjacent sandwiches.

### 2.2 Organic Cocrystal Systems

In the application of direct-space structure solution methods, the presence of more than one molecular fragment in the asymmetric unit [26,27] makes the problem more complex both in terms of the number of degrees of freedom (ie. the number of structural parameters varied to generate new trial crystal structures), and to a certain extent, the effect on *R*-factor discrimination. There are a few examples of such materials solved from powder diffraction data using the direct-space structure solution approach, a situation made more complicated due to the presence of two entirely different entities in the cocrystal with the location of each molecule in the unit cell being unique and non-superimposable.

**Scheme 2:**
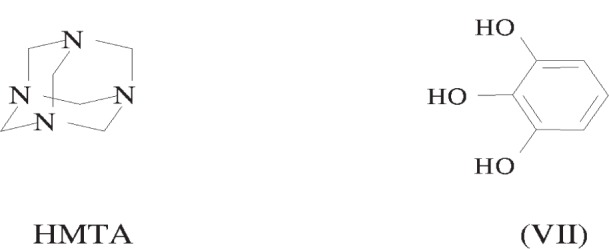


Previous studies have used single-crystal x-ray diffraction to explore the use of bis- and trisphenols in crystal engineering and the interaction of this type of phenol, acting as a hydrogen bond donor, with hexamethylenetetramine, (CH_2_)_6_N_4_ (HMTA), as a hydrogen bond acceptor [28]. However in the case of the 1:1 adduct of 1,2,3-trihydroxybenzene (pyrogallol, VII) and HMTA (shown in Scheme 2), investigation of the crystal structure has been carried out using powder diffraction data obtained from a conventional laboratory-based diffractometer [6].

#### 2.2.1 Structure Determination of Pyrogallol-HMTA (1/1)

The powder diffraction pattern was indexed giving a monoclinic unit cell and space group consistent with the presence of one molecule of each component in the asymmetric unit. The structural model used in the Monte Carlo structure solution comprised a complete HMTA molecule and a pyrogallol molecule excluding the hydrogen atoms on the three hydroxyl groups. Both these molecules were constructed using standard bond lengths and angles and treated as rigid bodies in the calculation. Trial structures were generated by translation and rotation of both molecules completely independently of each other within the unit cell. With more than one independent molecule required to define the structure, the number of degrees of freedom required for random movement is increased (from 6 to 12 in this case) without conformational flexibility being introduced. The only additional constraint is a limit on the closest approach between the two independent bodies in the form of an artificially biased agreement factor.

**Fig. 2:**
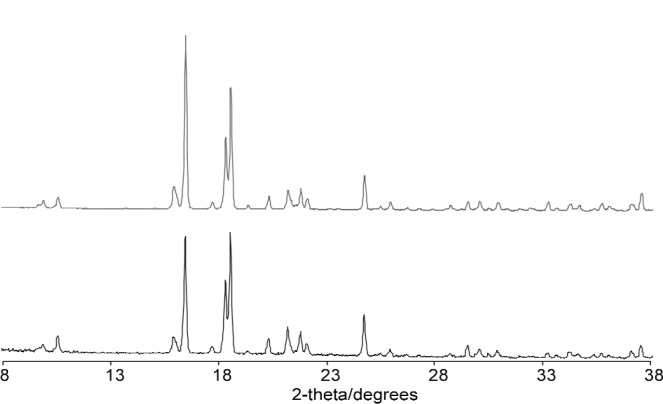
X-ray powder data for pyrogallol-HMTA (1/1): Capillary data (top) and disc data (bottom).

**Fig. 3:**
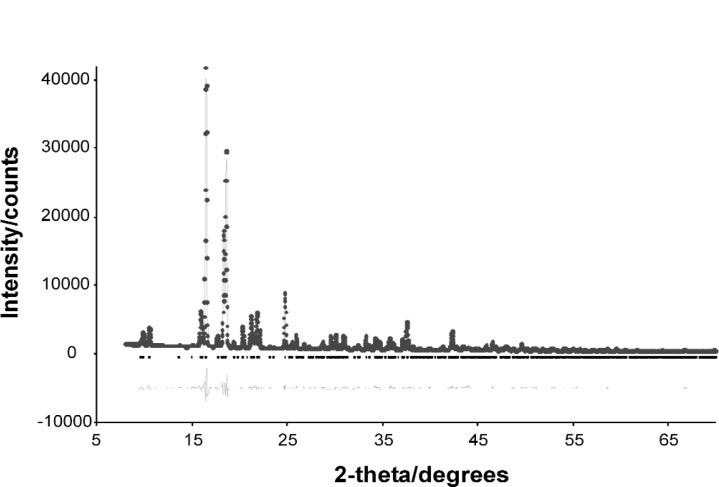
Final observed (circles), calculated (solid line) and difference (below) powder x-ray diffraction profile for the final Rietveld refinement of pyrogallol-HMTA (1/1). Reflection positions are also marked.

The best structure from the Monte Carlo calculation was used as the starting model for Rietveld refinement and the positions of all atoms refined subject to soft restraints on the standard geometric parameters (Table 1). As in the previous structure, isotropic atomic displacement parameters were refined for the non-hydrogen atoms only, and constrained according to atom type or environment. Diffraction data had been collected with the sample packed in both disc and capillary geometries and it was clear from the difference in relative intensities of related peaks in these data that there was a significant degree of preferred orientation present (Fig. 2). Although the effects of the preferred orientation were minimised by use of the capillary data set for both solution and refinement, variation of a preferred orientation parameter in the [100] direction was required (Table 1). A plot of the final Rietveld refinement for this structure is shown in Fig. 3. The hydroxyl H atoms were placed in positions calculated from the coordinates of the hydrogen-bond donor and acceptors, but were not included in the refinement.

#### 2.2.2 Hydrogen Bonding and Molecular Packing

**Fig. 4:**
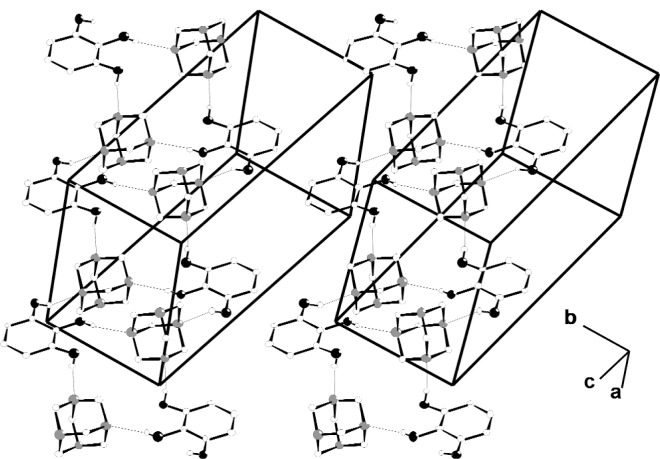
Stereoview of the crystal structure of pyrogallol-HMTA (1/1) showing the alternating O–H⋯N hydrogen-bonded rings running parallel to [100]. Hydrogen atoms involved in hydrogen bonding are shown as small open circles, and hydrogen bonds represented by thin lines.

All three hydroxyl groups in the pyrogallol molecule act as hydrogen bond donors with three N atoms each from different HMTA molecules acting as acceptors. This differs from the majority of systems in which HMTA generally acts as a double acceptor of hydrogen bonds [28]. Rather less frequently, HMTA behaves as an acceptor of just one hydrogen bond [28,29], a full complement of four hydrogen bonds, or as in this case, of three hydrogen bonds [30–32]. O–H⋯N hydrogen bonds are formed from the hydroxyl groups in the 1 and 3 positions linking alternating pyrogallol and HMTA molecules in a chain running parallel to the [100] direction. Pairs of these chains are linked by further O–H⋯N hydrogen bonds from the hydroxyl groups in the 2 positions to another N atom in each HMTA unit forming two distinct cyclic R^4^_4_(18) motifs. The result is a lightly-puckered molecular ribbon running parallel to the [100] direction in which the HMTA cages lie alternately above and below the plane (Fig. 4).

**Fig. 5:**
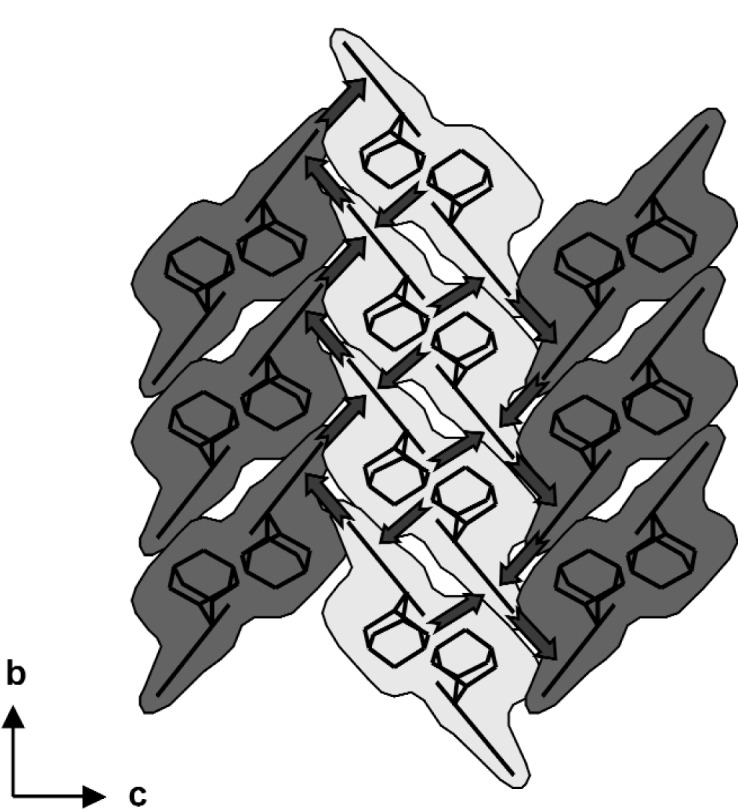
A schematic diagram of the crystal structure of pyrogallol-HTMA (1/1) viewed down the *a*-axis with the molecular ribbons shown end-on. Each ribbon is represented by a shaded area with the planar pyrogallol units and HMTA cages indicated by black lines. C–H⋯π(arene) interactions are indicated by block arrows.

These ribbons are linked into a continuous three-dimensional framework by C–H⋯⋅π(arene) interactions. There are edge-to-face interactions between pyrogallol units in neighbouring ribbons, occupying one face of each ring: the other face of each ring is involved in a C–H⋯π(arene) interaction with a C–H bond from an HMTA unit in a neighbouring ribbon. The latter C–H⋯π(arene) interactions link sets of neighbouring parallel ribbons into columns stacked in the [010] direction, while those between the pyrogallol units link neighbouring stacks together to form a herringbone pattern (Fig. 5). Propagation of these two types of C–H⋯π(arene) interactions based on aromatic and aliphatic C–H bonds respectively links all the parallel ribbons into a single bundle, so that the overall supramolecular structure is three-dimensional.

### 2.3 Nicotinic Acid Derivatives

**Scheme 3:**
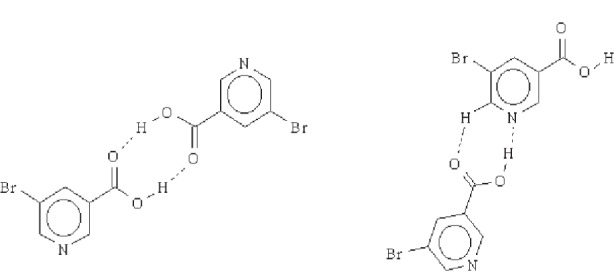


5-Bromonicotinic acid is a relatively simple molecule that can, in principle, provide important information about competition between intermolecular forces since it has limited conformational flexibility and there are relatively few primary supramolecular assemblies that can be envisaged (Scheme 3). This compound suffers from poor crystal growth, and attempts at recrystallization resulted only in the formation of a range of solvates. The structures of three of these solvates (with ethylacetate, acetonitrile and methanol) were all obtained from single-crystal data, whereas the crystal structure of the parent compound itself has been solved from powder diffraction data using the Monte Carlo technique [8].

#### 2.3.1 Structure Determination of 5-Bromonicotinic Acid

The powder diffraction pattern was indexed giving a unit cell and space group consistent with one molecule in the asymmetric unit (unlike the solvate structures with multiple parent, and often multiple solvate molecules in the asymmetric unit). The structural model used in the Monte Carlo calculation comprised the complete molecule excluding the carboxylic hydrogen, and was constructed using standard bond lengths and angles. The pyridine ring was assumed to be planar (as in similar systems) and the molecule treated as a rigid body in the structure solution, with the pyridine and carboxylic acid groups constrained to be coplanar.

**Fig. 6:**
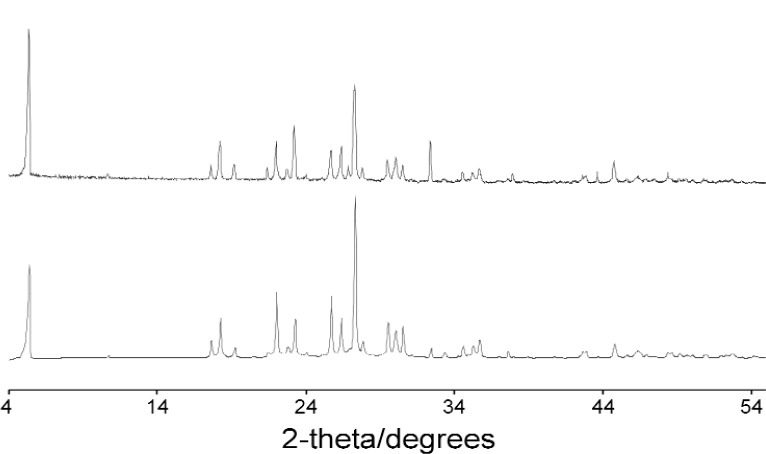
X-ray powder data for 5-bromonicotinic acid: Disc data (top) and capillary data (bottom).

In the Monte Carlo structure solution, the structural model was rotated and translated within the unit cell from an initial random location. The best solution found in the structure solution calculation was taken as the starting model for Rietveld refinement (Table 1), but after several cycles it was clear that the model had become significantly distorted. The data set used for indexing and structure solution had been collected using a stationary disc. Comparison of this data with a second data set collected using capillary geometry, showed the presence of a high degree of preferred orientation (Fig. 6), possibly accounting for this distortion in molecular geometry. A second Monte Carlo calculation was carried out using the capillary data set, under the same optimization conditions as above; this generated the same structure solution but with a better fit to the profile (Table 1). The structure was then refined successfully using this data set, with all atom positions refined subject to soft constraints on standard geometry. Variation of a preferred orientation parameter along the [010] direction was still required in refinement, and isotropic atomic displacement parameters refined for non-hydrogen atoms only and constrained according to atom type. The carboxyl hydrogen atom was placed in a position calculated from the coordinates of the donor and acceptor carboxyl oxygen atoms, but had no effect on the refinement.

#### 2.3.2 Hydrogen Bonding and Molecular Packing

**Fig. 7:**
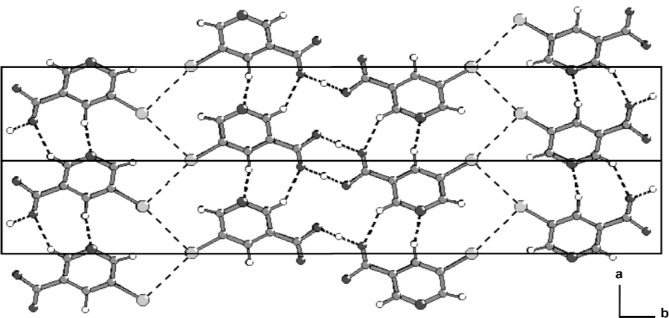
The crystal structure of 5-bromonicotinic acid showing the hydrogen bonds and Br⋯Br interactions (as dotted lines) resulting in an infinite layer of molecules.

The crystal structure of 5-bromonicotinic acid differs significantly from the solvate structures. The molecules of the parent compound form centrosymmetric dimers through a self-complementary acid-acid hydrogen-bond motif, rather than formation of the dominating C–H⋯O and O–H⋯N supramolecular interactions and infinite chain motif found in the solvates. Adjacent acid dimers are connected into antiparallel ribbons by C–H⋯O and C–H⋯N hydrogen bonds. These infinite planar ribbons run parallel to the [100] direction and are arranged into two-dimensional sheets held together by weak Br⋯Br interactions (Fig. 7), with π-π stacking of these layers to form a three-dimensional structure.

## 3. Polymorphism

The study of polymorphism in organic materials continues to attract considerable academic and industrial attention, but still requires full structural characterisation in each case to attain a true understanding of the aspects controlling this phenomenon. However, the conditions used to prepare many polymorphs, in particular metastable forms, often yield materials that occur only as polycrystalline powders. These systems are therefore often both initially identified and their structure investigated by powder diffraction alone [7].

### 3.1 A New Polymorph of Chlorothalonil

Chlorothalonil (2,4,5,6-tetrachloro-1,3-dicyanobenzene) is a broad-spectrum fungicide used to control fungi that threaten turf, vegetables, and other agricultural crops. A recent study has suggested that there may be three polymorphs of chlorothalonil [33] although only form I, the commercially available form, has been fully structurally characterized [34]. As a system reported to show possible polymorphic behaviour, chlorothalonil was chosen as a test for independent simultaneous studies involving an experimental search for new polymorphs and theoretical crystal structure prediction [35].

X-ray powder diffraction data was used both initially to confirm the preparation of a new polymorph (form II), obtained by recrystallization from butanol, and subsequently in the determination and rationalization of its crystal structure. In the event, the structure of form II is disordered, and so cannot be predicted by current theoretical methods.

#### 3.1.1 Structure Determination of Chlorothalonil Form II

**Fig. 8:**
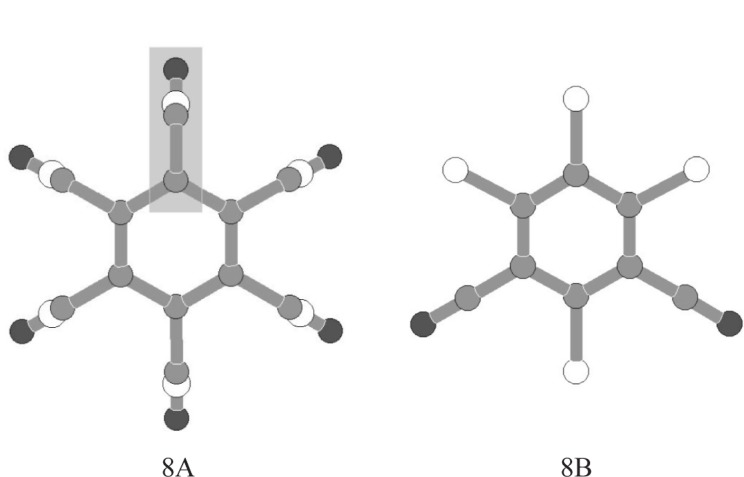
Structural models of the chlorothalonil molecule used in structure solution of (a) *R*-3*m* disordered structure (a C-C-Cl-N spur is indicated); (b) *P*1 ordered structure.

The powder diffraction pattern of form II was indexed on the basis of the first 21 observable peaks using the CRYSFIRE package [36]. A large number of cells were obtained with high figures of merit (*M*_20_ > 250) [37], although many of these cells did not satisfy suitable density requirements (often with a volume less than that required for a single chlorothalonil molecule). Despite having a relatively low figure of merit (*M*_20_ = 41), the unit cell chosen was that of highest symmetry; a hexagonal unit cell *a* = *b* = 9.24 Å, *c* = 10.10 Å with a volume 747 Å^3^ (*Z*=3). Systematic absences suggested *R*-3 (148) and *R*-3*m* (166) as probable space groups, although both would require six-fold symmetry in the molecule. This is possible if the molecule is assumed to be disordered with the -C≡N and -Cl substituents on the benzene ring being indistinguishable and represented by a C-(C≡N)/Cl “spur”. In addition to this structure solution calculation (using a disordered hexagonal model (Fig. 8a)), a second structure determination was attempted using an “ordered” model in *P*1 (Fig. 8b). By consideration of only the most basic crystallographic symmetry (*P*1), we hoped to obtain a good “direct-space” approximation to the disordered structure that would provide an insight into the nature of the disorder and enable straightforward comparison with any ordered structures obtained from the crystal structure prediction calculation.

#### 3.1.2 Structure Solution in *R*-3*m* (Disordered Structural Model)

Structure determination was attempted initially in *R*-3*m* due to the higher symmetry constraints imposed on this structure by the *R*-3*m* space group. Structure solution was carried out using a grid search technique by rotation of a C-(C≡N)/Cl spur with relevant disorder occupancies, around the 0,0,*z* axis in 1° steps and over the range 0 ≤ *z* ≤ 0.5 at intervals of 0.1, thus generating a complete disordered molecular model (Fig. 8a). The best structure solution (that with the lowest *R*_wp_), with the chlorothalonil molecule lying parallel to the *ab* plane with atoms in the 2*x*,*x*,-*z* positions, was taken as the starting model for Rietveld refinement. The positions of all atoms were refined subject to symmetry and geometrical restraints, and refinement of a preferred orientation parameter was also required in the [001] direction (see Sec. 3.1.4). The final Rietveld refinement agreement factors are given in Table 1. This disordered structure was later confirmed by single-crystal x-ray diffraction studies.

#### 3.1.3 Structure Solution in *P*1 (Ordered Structural Model)

**Fig. 9:**
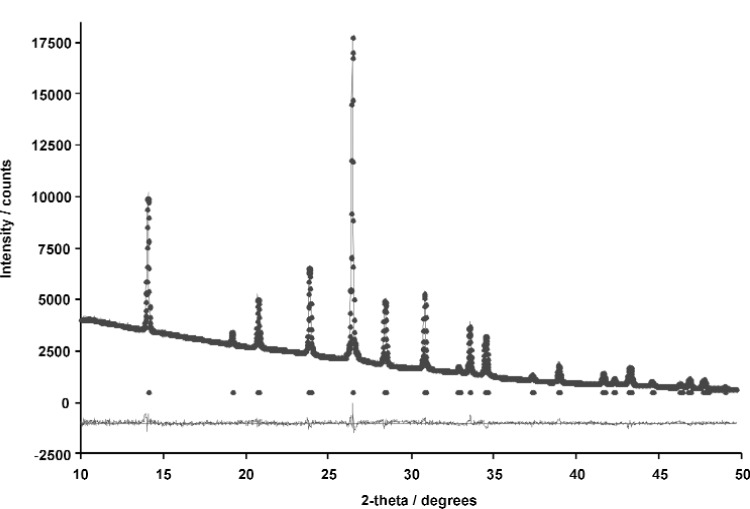
Final observed (circles), calculated (solid line) and difference (below) powder diffraction profile for the final Rietveld refinement of chlorothalonil in *P*1. Reflection positions are also marked.

The rhombohedral setting equivalent to the indexed hexagonal cell was used as a basis for the triclinic lattice parameters (a ≠ b ≠ c ≈ 6.32 Å, α ≠ β ≠ γ ≈ 94.2°) with one molecule in the asymmetric unit. The structural model used in the Monte Carlo structure solution calculation comprised the complete ordered molecule (Fig. 8b) constructed using standard bond lengths and angles. In the generation of trial structures, the chlorothalonil molecule was treated as a rigid body with only variation of the orientation of the molecule in the unit cell being required from a random initial position. A few structures were located with an *R*_wp_ value similar to that of the best structure (with the lowest *R*_wp_), but were related by 60° rotation of the model within the plane of the molecule. The best structure was taken as the starting model for Rietveld refinement and the positions of all atoms refined subject to soft geometrical restraints on standard geometry. Isotropic atomic displacement parameters were refined, but constrained according to atom type or environment. Variation of a preferred orientation parameter was also required in the [111] direction (see Sec. 3.1.4). The final Rietveld refinement agreement factors are given in Table 1 and the Rietveld plot shown in Fig. 9.

#### 3.1.4 Preferred Orientation Considerations

**Fig. 10:**
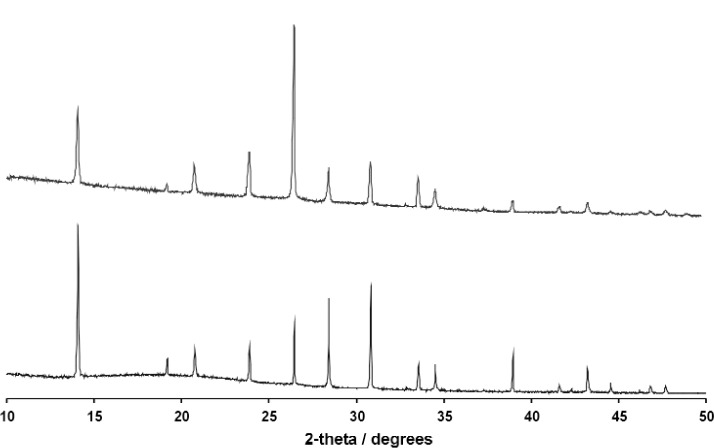
X-ray powder diffraction data for chlorothalonil form II: Capillary data (top) and disc data (bottom).

Initial attempts at structure solution in both *R*-3*m* and *P*1 generated structure solutions that despite having relatively low *R*_wp_ values (e.g., in the *P*1 calculation, the best structure solution had *R*_wp_ = 0.12, whereas the average range of values for a typical “wrong” structure was 0.22–0.24), were clearly incorrect and immediately rejected as implausible in terms of molecular packing. The rotation of a C-(C≡N)/Cl spur around a fixed axis in *R*-3*m*, or the movement of a single rigid molecule in *P*1 is a simple global optimisation problem, and hence the presence of preferred orientation in the data was investigated as a possible reason for unsuccessful structure solution. Both structure solution calculations were initially attempted using powder data collected in a flat disc. Subsequent collection of a data set using capillary geometry, and comparison with the original disc data clearly shows that the degree of preferred orientation present in this case is severe (Fig. 10). Consequently structure solution and refinement was only successful when carried out using the capillary data to minimize the preferred orientation effects of the plate-like crystallites, although a preferred orientation correction was still required in refinement.

#### 3.1.5 Molecular Packing and Comparison of Structures

**Fig. 11:**
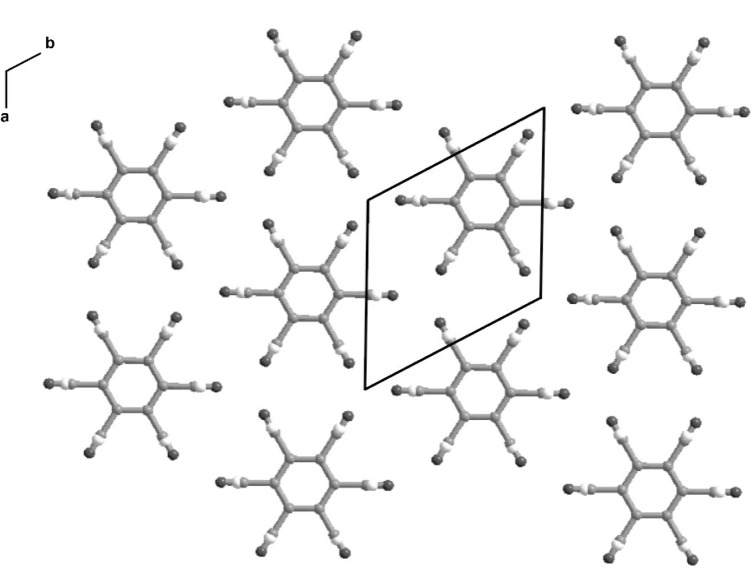
The *R*-3*m* disordered crystal structure of chlorothalonil form II showing a single layer in the (110) plane.

**Fig. 12:**
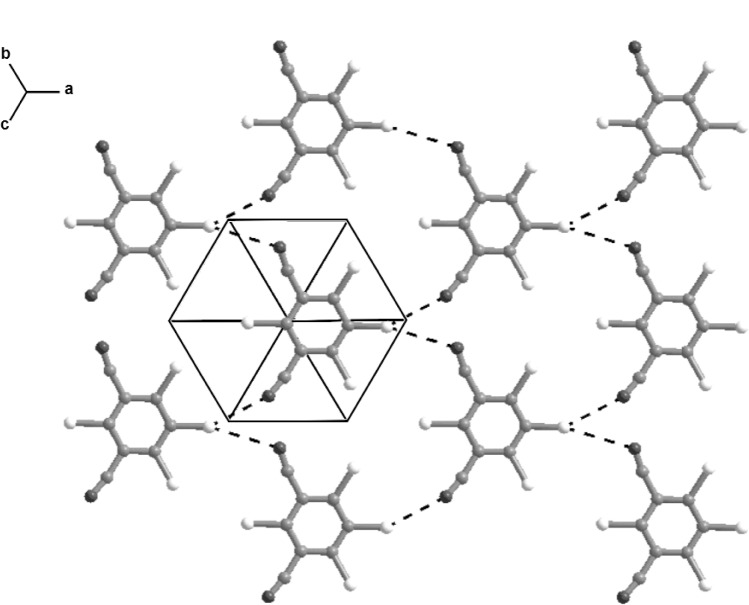
The *P*1 ordered crystal structure of chlorothalonil form II showing a single layer in the (111) plane. N⋯Cl interactions are shown by dashed lines.

The lack of any strong intermolecular bond functionality means that the molecular packing in chlorothalonil is controlled primarily by weak C≡N⋯Cl interactions. The *R*-3*m* and *P*1 crystal structures of form II are very similar in terms of molecular packing (Figs. 11 and 12), and differ only in the application of a disordered or ordered structural model (although the triclinic structure is only an ordered approximation to the true disordered structure). Both structures consist of infinite planar sheets in which the molecules are held together by C≡N⋯Cl interactions, with π-π stacking of these layers to form a three-dimensional structure.

In the *R*-3*m* structure, each molecule is surrounded by six others in each sheet with an N⋯Cl distance of 3.272(6) Å (Fig. 11). These sheets run parallel to the (001) plane with an inter-layer distance of 3.364(6) Å. A similar inter-layer distance of 3.36(2) Å is found in the *P*1 structure, although the sheets lie in the [111] direction, and the molecules in each sheet are linked by N⋯Cl interactions of 3.05(4) Å and 3.35(3) Å (Fig. 12).

However, it is clear that in the *P*1 structure the intermolecular distance between the cyano groups in neighbouring molecules in the [011] direction is too short (2.45(4) Å). Rotation of the molecule in 60° steps within the (111) plane results in similar molecular packing with close cyano contacts running between molecules in the [101] or [110] directions, respectively. The five new crystal structures generated by these rotations are also indistinguishable by *R*_wp_ (calculated from the experimental powder data), confirming that any of the six orientations give an equivalent representation of the disordered structure. As the *P*1 structure is clearly implausible in terms of intermolecular packing, we can conclude that the disorder in this system does not arise through the existence of domains in the crystal each containing a section of the *P*1 symmetry structure rotated through all six possible orientations, but may still be a reasonable approximation to the true crystal structure through correlated disorder.

## 4. Concluding Remarks

The ability to determine the crystal structures of small organic materials that suffer from poor crystal growth is essential if reliable conclusions are to be drawn from systematic structural studies of intermolecular forces. Many of these structures show, not surprisingly, crystal packing that is atypical, but play a key role in our understanding of non-covalent interactions.

In the case of 2,4,6-tri-isopropylbenzenesulfonamide, the initial room-temperature dataset collected using a laboratory x-ray source could be indexed, but the structure could not be determined from these data using traditional structure solution methods. The success achieved with low-temperature synchrotron data raises the possibility that the previous attempt at structure solution may have been hampered by the occurrence of intramolecular rotations at room temperature. While rotation of the sulfonamido group about the C-S bond is unlikely because of the hydrogen bonding, rotation of the isopropyl groups about the C(aryl)- CHMe_2_ bonds seemed plausible. However, solid-state CP-MAS NMR investigations [38] have shown that such a rotation is not observed even at room temperature, and we conclude that it is a combination of the superior resolution of the synchrotron data and the application of improved structure solution software that has now permitted structure determination. Attempts at the structure solution of 2-toluenesulfonamide (IV) using the Monte Carlo method were unsuccessful. However, structure determination has been achieved recently by the application of another direct-space method based on the differential evolution algorithm [39], but using the original diffraction data, collected some ten years ago.

We have also demonstrated that conventional laboratory powder diffraction data, collected under non-ideal conditions (in which the sample displays significant preferred orientation) can be used to study such structures. The development of direct-space structure solution methods has had a significant impact in this area, and may prove to be more powerful than thought if shown to be robust when dealing with data that is distorted by preferred orientation. This is clearly illustrated by the structure determination of pyrogallol HMTA (1/1), in which despite the presence of two entirely different molecular components in the structure, and the evidence of preferred orientation in the data, structure solution and refinement ran smoothly.

Although the structure solution of the majority of materials described in this paper progressed in a relatively straightforward manner, the structure determination of chlorothalonil form II proved to be more problematic. Despite being a simple structural problem in terms of direct-space structure solution methodology, the presence of a severe degree of preferred orientation in the diffraction data resulted in the failure of initial attempts at structure solution. Given the earlier successes of the direct-space approach, this was somewhat unexpected (even though the preferred orientation in this case was much more severe). However, this clearly demonstrates that no matter how straightforward the structure may seem, measures should be taken in sample preparation or choice of data collection conditions to minimize these sample effects and ensure the best chance of success in structure solution. The advantages of using capillary data for the structure solution of “sheet-type” organic materials are obvious, although disc data can also be used. However, it is important to note that all the data used here were collected in transmission geometry and that detrimental sample effects are often maximized using “flat plate” reflection geometry which should be avoided if possible.

Such considerations enabled the determination of the structure of a new disordered polymorph of chlorothalonil, but attempts to rationalize this disorder using experimental data resulted in a low symmetry structure that was implausible in terms of crystal packing. Although the disorder in this structure cannot be predicted by current computational methods, our recent structure prediction studies have generated an alternative ordered layer structure that provides a valuable insight into the nature of the disorder, and demonstrates how the complementary use of these two techniques can reveal structural information that would be unavailable if the experimental and theoretical results were considered independently [35].

## Figures and Tables

**Table 1:** Crystal data, Monte Carlo structure solution parameters (number of parameters used to define structural model, number of Monte Carlo moves, typical *R*_wp_ for random “wrong” structures and *R*_wp_ for the best structure solution), and final Rietveld refinement agreement factors (including a preferred orientation fraction (PO))

Compound	2,4,6-triisopropyl benzenesulfonamide	Pyrogallol HMTA (1/1)	5-bromonicotinic acid	Chlorothalonil (disordered)	Chlorothalonil (ordered)
Crystal data					

Formula	C_15_H_25_NO_2_S	C_12_H_18_N_4_O_3_	C_6_H_4_BrNO_2_	C_8_Cl_4_N_2_	C_8_Cl_4_N_2_
*a* (Å)	16.9600(6)	10.7691(2)	5.1158(2)	9.2392(4)	6.3082(5)
*b* (Å)	8.1382(2)	7.0107(2)	33.159(2)	9.2392(4)	6.2995(4)
*c* (Å)	11.7810(2)	16.7519(4)	3.9500(1)	10.0969(5)	6.3137(5)
Alpha (°)	90	90	90	90	94.202(6)
Beta (°)	104.777(1)	91.402(2)	95.526(3)	90	94.059(4)
Gamma (°)	90	90	90	120	94.286(5)
Volume(Å^3^)	1572.3(1)	1264.38(3)	666.96(8)	746.4(1)	248.79(3)
Space group	*P*2_1_/*c*	*P*2_1_/*n*	*P*2_1_/*n*	*R*-3*m*	*P*1

Structure solution					

Parameters	10	12	6		3
MC moves	200000	500000	100000	1800	10000
Typical *R*_wp_	0.47–0.61	0.52–0.68	0.47–0.55	0.16–0.18	0.17–0.19
Best *R*_wp_	0.30	0.19	0.31 (disc),	0.06	0.07
			0.19 (capillary)		

Refinement					

Final *R*_wp_	0.070	0.074	0.119	0.038	0.033
Final *R*_p_	0.049	0.054	0.090	0.025	0.024
*χ*^2^	2.97	5.75	1.08	3.03	2.19
PO		0.807	1.147	0.882	0.924
